# Study of Attitudes and Practice of Physicians Regarding Consultation-Liaison Psychiatry in Teaching Hospitals of Mazandaran, Iran

**Published:** 2014

**Authors:** Mehran Zarghami, Samaneh Farnia, Ali-Reza Khalilian, Tahereh Amirian

**Affiliations:** 1 Professor, Psychiatry and Behavioral Sciences Research Center, Addiction Institute AND Department of Psychiatry, School of Medicine, Mazandarn University of Medical Sciences, Sari, Iran.; 2 Assistant Professor, Psychiatry and Behavioral Sciences Research Center, Addiction Institute AND Department of Psychiatry, School of Medicine, Mazandarn University of Medical Sciences, Sari, Iran.; 3 Professor, Psychiatry and Behavioral Sciences Research Center, Addiction Institute AND Department of Public Health, Mazandarn University of Medical Sciences. Sari, Iran.; 4 Researcher, Psychiatry and Behavioral Sciences Research Center, Addiction Institute, Mazandarn University of Medical Sciences, Sari, Iran.

**Keywords:** Attitude, Consultation-Liaison Psychiatry, Practice

## Abstract

**Objective**: Consultation-liaison (CL) psychiatry interfaces between psychiatry and other medical disciplines to promote integrated care of patients. The purpose of this study is evaluation of attitudes and practice of Mazandaran University of Medical Sciences physicians of teaching hospitals regarding CL psychiatry.

**Methods:**In this descriptive study, all of the general practitioners, specialist and subspecialist physicians and assistants working in teaching hospitals of Mazandaran University of Medical Sciences were requested to fill in a questionnaire which was designed based on previous studies and observations to assess their attitudes and practice. Data were analysed by SPSS-16 software, using chi square.

**Results: **One hundred and forty nine (62.6%) physicians had very positive attitudes and 89 cases (37.4%) had positive attitudes; 234 physicians (98.3%) had acceptable practice, and 4 cases (1.7%) had unacceptable practice. There were no significant differences between physicians with positive and very positive attitudes and between physicians with acceptable and unacceptable practice regarding gender, age, education, specialty and place of work (hospital). The most common reasons of physicians for not requesting psychiatric consultation were lack of time, forgetfulness, lack of access to psychiatrist, and lack of belief in the need for psychiatric consultation respectively.

**Conclusion**: The findings of this study indicate the successful psychiatric educations and psychiatrists practice in formation of positive attitudes and acceptable practice regarding CL Psychiatry in these university hospitals. No significant differences between different specialties and work place hospitals indicate that they are similarly affected.

## Introduction

Psychiatry has been described as the oldest technique and newest knowledge in medical sciences (1). Although, in eastern approach, the body was not separated from the spirit and the spirit was not separated from the body, and pioneers such as Avicenna had used the mutual effect of mind on body in treatment of their patients (2), in Western's literature some physicians such as, Benjamin Rush, William Osler, Adolf Meyer, and Franklin Ebaugh had used psychiatric interventions for patients with medical problems in modern medicine. Henry entered psychiatrists for educational purposes in the public hospitals in 1929 (3). Nowadays, consultant-liaison (CL) psychiatry work as an interface between psychiatry and other medical disciplines. In addition, psychiatrists evaluate the interaction between biological and psychological phenomena (4).

Psychiatric aspects have been demonstrated as effective factors developing and predicting general medical diseases (5, 6). For example, the effects of depression and stress on impairment of immune system, increasing platelets aggregation and inducing cardiac arrhythmias or ischemia have been demonstrated (5).

In the other hand, comorbidity of somatic diseases and psychiatric disorders increases the medical costs and impairs the patients' function. Overall, quality of psychiatric services is weak at the primary health care (5, 7). For example, although depression, anxiety and somatization are diagnosed in 50% of the cases of primary care, such diagnoses do not necessarily lead to appropriate treatment (5). Currently, the main reasons of referring the patients to the psychiatrist include assessment of mental status and function, the possibility of depression, a history of psychiatric disorder, anxiety and substance use disorders (8-10).

In the past two decades, the number of CL psychiatrists has been increased in many countries. As the patients with comorbidity of medical and psychiatric disorders, need to receive high quality attention and treatment in all levels, obtaining especial standards is necessary for CL psychiatry (8). Training of staff and specialists would be necessary to increase familiarity with psychiatric disorders and psychosomatic diseases.

The aim of the current study was to evaluate the physicians’ attitude and their practice in educational hospitals of Mazandaran University of Medical Sciences (MAZUMS) in the north of Iran, regarding CL psychiatry.

## Materials and Methods

This research was a descriptive study. All physicians working in educational hospitals of MAZUMS in Sari and Qaemshahr were asked to participate to this research from May to August 2011. Exclusion criteria was unwillingness to respond the questionnaire or giving contradictory responses to pair questions of “2 and 7”, “20 and 7”, “5 and 6”, and“16 and 15”. 

The research instrument was a questionnaire that was designed based on previous observations and studies to assess physicians' attitudes and performance. The questionnaire included 29 closed ended questions to check the attitude. Their scores were based on a Likert type scale of 5 degrees (between -2 to 2): “I completely agree” scored 2, “I agree” scored 1, “I have no comment” scored” 0, “I disagree” scred-1, and “I completely disagree” scored-2. Total scores of 30 to 58 were labeled “very positive attitude”, 0 to 29 were labeled “positive attitude”, 0 to -29 were labeled “negative attitude”, and -30 to -58 were labeled “extremely negative attitude”. A number of open ended questions were added at the end of the questionnaire to evaluate the practice. Practice questions were scored from 0 to 10; a score of 0 to 5 assumed “unacceptable” practices and 6 to 10 were assumed “acceptable”.

After confirming the face and content validity of the questionnaires by the members of Department of Psychiatry and Psychiatry and behavioral sciences Research Center of Mazandaran University of Medical Sciences and Iranian Psychiatric Association (Mazandaran Branch), 19 physicians filled the questionnaire by two weeks interval to evaluate test-retest reliability of the questionnaire. Correlation coefficient was 0.83. Measuring the internal consistency of the questions, Cronbach's alpha coefficient was 0.91. After oral description of the aims, methods and confidentiality considerations of the study, questionnaires were manually distributed in Clinics or doctor's offices and the pavilion of General Practitioners, Residents, specialists and subspecialists (except psychiatrists and psychiatric residents) working in Imam Khomeini, Avicenna, Zare, Fatemeh Alzahra Hospitals of Sari and Razi of Quaemshahr, and after filling, they were manually collected.

Data were analyzed by SPSS-16 software, using chi square. 

## Results

All of the study populations (260 physicians) were invited to participate in this descriptive study. Among participants, 34 were general physicians, 103 were residents, 2 were fellows, 74 were specialist and 47 were subspecialists. Fourteen physicians were not satisfied to participate in this project, and 8 participants were excluded because of their inconsistent responses. A total of 238 physicians working in educational hospitals of Sari and Qaemshahr were evaluated.

Average age of the population was 37.61 ± 5.3 years. Among these, 144 physicians were male (60.5%) and other 94 cases were female (62.6%). Totally, 149 cases (62.6%) had very positive and 89 cases (37.4%) had positive altitudes. Also, 234 cases (98.3%) and 4 cases (1.7%) had acceptable and unacceptable practices, respectively. There were no significant differences between sex of individuals with very positive and positive attitudes (p < 0.25) and acceptable and unacceptable practices (p < 0.55). Also, there were no meaningful differences between age of participants with very positive and positive altitudes (p < 0.18) and acceptable and unacceptable practices (p < 0.38). The differences between individuals with very positive and positive altitudes (p < 0.33) and acceptable and unacceptable practices (p < 0.40) with respect to their education level and academic fields were not significant too. Also there were no significant differences between those with a positive and very positive attitude (p < 0.24) and acceptable and unacceptable practice (p < 0.51) in terms of their work place (hospitals). Half of the participants (130 physicians) sometimes requested psychiatric consultation for their patients. In the cases which they did not request psychiatric consultation, 18 individuals believed that there is no necessity to ask for consultation, 68 participants stated that they forgot to ask for it, 114 cases claimed that they do not have time and 37 participants mentioned that they did not request a consultation because of lack of appropriate access to the psychiatrists. 

In response to the question” During explanation of unfavorable course of disease for patients, have you ever been helped by a psychiatrist?”, 148 participants gave positive answer and 90 persons gave negative answer.

In evaluation of physicians’ views and reactions about drug-dependent inpatients, 229 doctors requested psychiatric consultation and 9 physicians did the intervention themselves.

In facing with a patient whose signs are not explained by a general medical condition, adverse drug reactions or substance abuse, 4 physicians mentioned that “they assure patients that they have no problem”, 2 physicians said that they “use suggestion for removing symptoms”, 25 doctors recommended antidepressants or sedative hypnotic agents, and 207 physicians requested psychiatric consultation.

About patients who committed suicide, 11 physicians released patients after solving their medical problems, 5 doctors referred the outpatients to psychiatrist and 222 requested emergency psychiatric consultation.

## Discussion

Accessibility of mental health services is necessary for all patients (either inpatient or outpatient). About 30 to 60 percent of inpatients of non-psychiatric wards have comorbid psychiatric disorder (8, 11-13). Unfortunately, only 30 to 50 percent of them are detected (14-17), and psychiatric consultation is requested for 1.14 to 3.7 percent of them (18-20). The need for psychiatric services in certain groups such as emergency and intensive care units, as well as cancer and organ transplant patients and patients with chronic diseases are more apparent (21). Despite the prevalence of psychiatric disorders in some wards such as dialysis, radiation therapy and intensive care units (22-24), and although these comorbidities would lead to prolonged hospitalization and increased medical costs as well as disabilities and mortalities (25, 26), only a few psychiatric consultations are requested (20, 24).

Some studies concluded that despite acceptable and good knowledge and attitudes of non-psychiatric specialists and subspecialists, it is necessary to pay attention to their weak practice for disorders such as depression (20). In this research, most of physicians recognized social and psychological aspects as important factors of the diseases. So the presence of a resident psychiatrist in public hospitals is necessary. Majority of physicians believed that the presence of CL psychiatrist in ward rounds and morning reports is also necessary, and psychiatric consultation is beneficial for management of surgical patients and dealing with substance dependent patients. 

There are significant variations in the characteristics of patients referred to CL services in different countries, especially in the field of deliberate self-harm and drug abuse. For example these services in Germany don’t see such patients, although in other European countries these patients constituted one-quarter to one-third of the patients referred to CL psychiatry/psychosomatic services (27). Most of the physicians in the current study requested psychiatric consultation for substance dependent patients or patients who have committed suicide, or when they cannot find a medical cause for the disease. In the Morgan et al.’s study, most of physicians and surgeons of a hospital in London believed that psychological factors can affect prognosis of medical diseases and these factors must be assessed routinely. They believed that this assessment must be more carefully done for dying and poisoned patients (6).

Presence of psychiatrist in centers for treatment of patients with cancer or chronic diseases or organ transplanted patients is necessary in many countries, and absence of them causes the performance of these centers to be unacceptable in the view of legal and government authorities. Most non-psychiatric specialists emphasize on the necessity of psychiatrists’ cooperation in management of these patients (21). Nevertheless, many hospitals do not provide regular and fulltime psychiatric services (21). Recently the ministry of health, treatment and medical education of Iran has included the presence of psychiatrists as an indicator of promotion of hospitals’ grading. 

Wide differences occur among physicians and nurses in attitudes towards CL psychiatry (28). A previous study has shown that acceptance of unlimited access to CL psychiatric services from nurses, other paramedical staff and junior doctors in addition to senior medical staff, would be resulted in an increase in referral rate and a significant alteration in the types of problem attracting referral (29). Participants in the current study recognized that psychiatrists are effective on coping of the care giver staff with patients, and believed that the bio–psycho-socio–spiritual approach of the diseases should not be limited to psychiatric disorders. Most of the physicians had tendency to request psychiatric consultation. In the case of non-requesting consultation, they considered lack of time, forgetting and lack of access to psychiatrists as the most important causes respectively.

A previous study has shown shortcomings in the amount of experience of the psychiatrists who performed the consultations, in the tone, readability, and organization of the reports provided by the consultants and in the timing of the consultations, as well as in follow-up of patients. Resolving these problems will consequently lead to increased use of the CL service and improves the quality of psychiatric referrals (30). Psychiatric department of Mazandaran University of Medical Sciences had started educational, research and treatment activities at this university over two decades. Undoubtedly, it has influenced physicians’ attitudes and practices of these hospitals. Consultation feedbacks gradually shape physicians’ attitude and function. If physicians take positive feedback from psychiatric consultations, they will request more consultations for next patients, and if they do not take positive feedback from consultant, negative attitude and practice will be formed gradually. 

Although, it was not found other studies to survey physicians’ attitude and practice about CL psychiatry directly, consultations status in the other centers infers to lack of suitable attitude and practice in this context indirectly (8, 19, 21, 24, 31-33).

Physicians other than psychiatrists indicate less attention to psychosocial aspects of diseases, possibly due to lack of enough training during their educational courses. Establishment of CL psychiatry ward provides this opportunity for medical students to become more familiar with bio-psycho-socio-spiritual nature of diseases, and consequently to have better skills for screening of psychiatric problems in medical diseases and doctor -patient relationship. 

Some authors believe that CL psychiatrists must commit tasks more than clinical descriptions and assessments to play more roles in educating physicians (27). Indeed, CL psychiatrists provide information to their non-psychiatrist colleagues via meetings and workshops other than consultations. This information significantly helps to provide better services by physicians and causes more familiarity with psychiatric aspects of diseases and increases the number of requested consultations. 

The findings of this study indicate the successful psychiatric educations and psychiatrists practice in formation of positive attitudes and acceptable practice regarding CL Psychiatry in Mazandaran University of Medical Sciences hospitals in north of Iran. No significant differences between different specialties and work place hospitals indicate that they are similarly affected. 

For more carefully surveying practice it is better to screen psychiatric disorders in non-psychiatric wards and then assess physicians’ function in respect of adequate management and /or performing psychiatric consultations.

## Footenote

**Declaration of interest:** None.
